# Rabies in the Caribbean: A Situational Analysis and Historic Review

**DOI:** 10.3390/tropicalmed3030089

**Published:** 2018-08-20

**Authors:** Janine F. R. Seetahal, Alexandra Vokaty, Marco A. N. Vigilato, Christine V. F. Carrington, Jennifer Pradel, Bowen Louison, Astrid Van Sauers, Rohini Roopnarine, Jusayma C. González Arrebato, Max F. Millien, Colin James, Charles E. Rupprecht

**Affiliations:** 1 Department of Preclinical Sciences, Faculty of Medical Sciences, The University of the West Indies, St. Augustine, Trinidad and Tobago; christine.carrington@sta.uwi.edu; 2 Pan American Health Organization (PAHO/WHO), Trinidad and Tobago Country Office, St. Clair, Trinidad and Tobago; vokatyal@paho.org; 3 Zoonosis Group, Pan American Foot-and-Mouth Disease Center (PANAFTOSA), Pan American Health Organization (PAHO/WHO), Rio de Janeiro, Brazil; vigilato@paho.org; 4 CIRAD, UMR ASTRE, Petit-Bourg, Guadeloupe F-97170, France; jennifer.pradel@cirad.fr; 5 ASTRE, CIRAD, INRA, Univ Montpellier, Montpellier F-34398, France; 6 Veterinary and Livestock Division, Ministry of Agriculture, Ministerial Complex, Tanteen, St. George’s, Grenada; bowen.louison88@gmail.com; 7 Ministry of Agriculture, Animal husbandry and Health and Fisheries, Paramaribo, Suriname; astrid_vs@yahoo.com; 8 St. George’s University, St. George’s, Grenada; rroopnarine@sgu.edu; 9 Ministerio de Salud Pública, La Habana, Cuba; yusaymacg@infomed.sld.cu; 10 Ministry of Agriculture, Natural Resources and Rural Development, Port-au-Prince, Haiti; maxfrancoismillien@gmail.com; 11 National Health Surveillance Unit, Ministry of Health, Georgetown, Guyana; hogancoli@yahoo.co.uk; 12 LYSSA LLC, Cumming, GA 30040, USA; charleserupprechtii@gmail.com

**Keywords:** Americas, Caribbean, canine rabies, Lyssavirus, mongoose rabies, bat rabies, vampire bat, zoonosis, CaribVET

## Abstract

Rabies virus is the only Lyssavirus species found in the Americas. In discussions about rabies, Latin America and the Caribbean are often grouped together. Our study aimed to independently analyse the rabies situation in the Caribbean and examine changes in rabies spatiotemporal epidemiology. A questionnaire was administered to the 33 member countries and territories of the Caribbean Animal Health Network (CaribVET) to collect current data, which was collated with a literature review. Rabies was endemic in ten Caribbean localities, with the dog, mongoose, and vampire bat identified as enzootic reservoirs. The majority of animal cases occurred in Puerto Rico, the Dominican Republic, and Haiti, while human cases only consistently occurred in the latter two areas. Rabies vaccination was conducted for high-risk animal populations with variable coverage, and rabies diagnostic capacities varied widely throughout the region. Illegal importation and natural migration of animals may facilitate the introduction of rabies virus variants into virus-naïve areas. Passive surveillance, together with enhanced methods and serological screening techniques, can therefore be of value. The insularity of the Caribbean makes it ideal for conducting pilot studies on reservoir host population management. Best practice guidelines developed for these reservoir hosts can be individually modified to the epidemiological status and available resources within each locality.

## 1. Introduction

Rabies is a highly virulent, globally distributed viral encephalitic zoonotic disease. The major etiological agent, rabies virus (RABV), belongs to the *Lyssavirus* genus [1] and is the only Lyssavirus species found in the Americas, where it circulates mainly in bats and mesocarnivores [2]. The virus is most commonly transmitted by bite exposure, with virus-laden saliva being introduced directly into the host through broken skin [3,4]. The clinical syndrome is variable with the disease presenting as either furious or paralytic [5,6]. Bat-transmitted rabies differs from canine-transmitted rabies, with the latter manifesting more encephalitic symptoms (hyperactivity and dysautonomia) typical of furious rabies, compared to the more peripheral symptoms (ascending flaccid paralysis) of the former [7]. Genetically, two major viral clades occur in the Caribbean: the cosmopolitan and the American clades [8]. The cosmopolitan clade consists of canine-maintained and canine-derived viruses, the latter resulting from spill-over and maintenance of canine-maintained lineages in neo-tropical mesocarnivores [9]. Canine-maintained viruses are disseminated as canine-transmitted rabies, which was first noted in the Americas during the 18th century [10] after being introduced during European colonization with the importation of domestic dogs [11]. Alternatively, phylogenetic, historical, and archaeological evidence suggests that the existence of bat-maintained rabies viruses, which falls within the American clade, predated European colonization of the New World [9,11,12,13]. Early reports indicate the presence of vampire bats that attacked Spanish colonists and likely fed on wildlife before the introduction of domestic livestock [14,15], which provided an abundant, readily-available resource for the expansion of vampire bat populations [16]. However, vampire bat-transmitted rabies in humans was only diagnosed during the beginning of the 20th century on the island of Trinidad [17], with later reports from countries in Central and South America [18,19,20]. With the concomitant decline in canine-transmitted rabies towards the end of the 20th century, these bats are now recognized as a major reservoir for RABV [20,21,22]. Rabies affects public, animal, and ecosystem health, and, as such, it is recognized as a key One Health issue [16]. With such issues, interventions are possible from many angles. However, in the case of rabies, intervention at the human health interface is unpredictable and very expensive; therefore, the most effective strategy is to target the animal source [16]. Prevention and control measures consequently mainly focus on the protection of susceptible animal populations through vaccination schemes, import control, and source reduction through reservoir animal population control.

In 1980, the annual average burden for canine rabies in Latin America and the Caribbean was approximately 25,000 cases in dogs and 350 cases in humans [23]. These high case numbers prompted the introduction of a regional Pan American Health Organization (PAHO) coordinated program for the control of canine-transmitted rabies in 1983 [23]. Since its implementation, there has been a dramatic decrease (>90%) in canine-transmitted rabies in both humans and dogs, with complete elimination in many countries [24,25] due to large-scale dog vaccination and population control, increased technical cooperation between ministries of health and agriculture, and improved access to pre-and post-exposure prophylaxis [26]. Despite these successes, which were hindered by political and economic constraints, the disease still exists in pockets in Latin America and the Caribbean (i.e. Bolivia, Brazil, Peru, Honduras, Guatemala, Haiti, Dominican Republic, and Cuba) [23,25]. As such, the action plan and expected elimination date have been updated several times, with the latest revision running up until 2022 [27]. The aforementioned regional program was launched at the first Meeting of National Rabies Program Directors in the Americas (REDIPRA), which is coordinated by PAHO and supported by numerous non-governmental organizations and public-private partnerships [28]. REDIPRA usually meets biennially to advocate for and review existing control strategies, compare program achievements against objectives, and update the regional action plan accordingly [27,28]. These meetings have been instrumental in the success of the regional program, aligning the countries towards a common goal and facilitating practical amendments to their national rabies control programs through shared experiences [29]. As part of the regional strategy, the Regional Information System for Epidemiological Surveillance of Rabies (SIRVERA), coordinated by the Pan American Foot-and-Mouth Disease Center (PANAFTOSA), PAHO, is an essential online database that facilitates monthly reporting of rabies for countries in the Americas. This system, which has been in operation since 1969, has recently been improved and is now a searchable database that facilitates accessibility to detailed information on reported cases such as diagnosis, viral variant, and aggressor animal species [30]. Although no information is collected on the type of surveillance method employed to capture cases, in Latin America and the Caribbean, as in other areas within the Americas, passive surveillance based on exposures and clinical suspicion may account for the majority of cases [31]. PAHO provides recommendations for active surveillance within canine populations [21]; however, they currently do not provide such recommendations for sylvatic reservoirs. Laboratory-based surveillance activities in Latin America and the Caribbean are supported by PAHO/WHO Research Centers in North America [32]. However, it has been recognized that there has been limited systematic evaluations of rabies surveillance carried out within the region [27,33].

Generally, in discussions about rabies in the Americas in the existing literature, Latin America and the Caribbean are often grouped as one area. However, due to limited surveillance and reporting, data available from most Caribbean countries is deficient and incomplete, whereas data from Latin America is usually more readily available. Hence, rabies data from the Caribbean is usually inadequately represented in the existing literature. Rabies is one of the priority diseases of the Caribbean Animal Health Network (CaribVET), which is a non-profit organization that involves the veterinary services from 34 Caribbean countries and territories, academic institutes, and regional and international organizations [34]. The network promotes collaborations and coordinated actions in the field of animal and veterinary public health to increase knowledge and improve the prevention and control of diseases in the Caribbean. This study, conducted by the Rabies Subgroup of the CaribVET Veterinary Public Health Working Group, therefore aims to present an analysis of the current rabies situation in the Caribbean to fill the existing gap in the literature and to examine changes in the spatio-temporal epidemiology of the disease since the 1930s.

## 2. Materials and Methods 

A questionnaire (see Supplementary Materials) was developed and administered by email during February 2014, to the veterinary and or public health authorities of the then-current 33 member countries or territories of CaribVET, through the Adobe Forms Central Web-based platform. The questionnaire was first validated and proof-read by the Rabies Subgroup of CaribVET and pilot tested by two Caribbean veterinary health officers from two CaribVET member countries chosen at random. Criteria for inclusion in the survey was all countries or territories that were current members of the CaribVET network. Data were collected between February 2014 and June 2015, during which time responses from 30 countries or territories were received. In analysing the responses, responses indicating ‘not sure’ were taken as ‘no’. Data were captured on (i) country rabies status, (ii) protocols and legislation, (iii) surveillance programs, (iv) rabies biologics, (v) reservoir population control programs, (vii) rabies diagnostics, and (vii) import health standards. Information collected by the survey was presented to and verified by the Steering Committee of CaribVET, which consists of Chief Veterinary Officers from member countries or territories and representatives of regional and international animal or public health institutions. These were then summarized and prepared as a descriptive analysis on the Caribbean rabies situation and supplemented by a literature review conducted on rabies within the rabies-endemic countries highlighted in the survey. The literature review was conducted on rabies in these countries from the first report of the disease to present. We used multiple electronic databases including PubMed, WHOLIS (World Health Organization Library Database), SciELO, ScienceDirect, and the AFPMB (Armed Forces Pest Management Board). Key words included ‘rabies’, ‘Caribbean’, ‘bat rabies’, ‘mongoose rabies’, ‘dog rabies’, ‘canine rabies’, and individual Caribbean country and territory names.

## 3. Results

### 3.1. Rabies Status and Main Animal Reservoir

**Figure 1:**
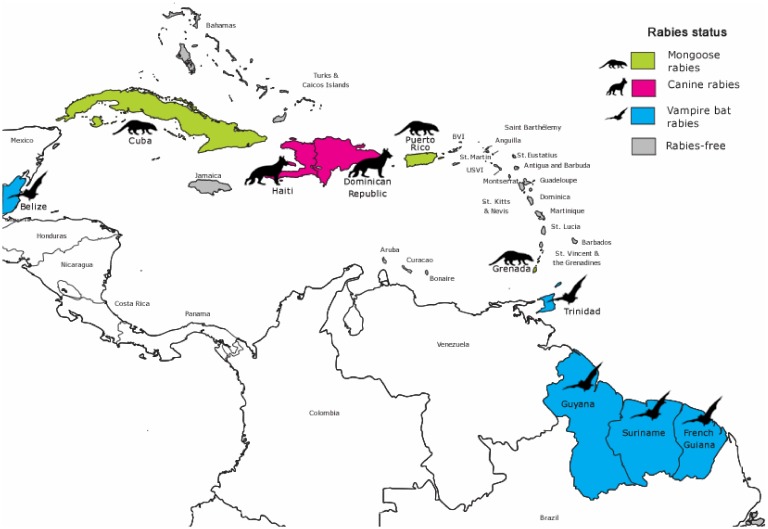
Main animal reservoirs for rabies virus and country endemic status in the Caribbean.

Rabies was reported to be endemic in ten Caribbean countries and territories, with the dog, mongoose, and vampire bat being the main enzootic reservoirs (Table 1 and Figure 1). On the island republic of Trinidad and Tobago, rabies was only reported to be endemic on the island of Trinidad. The other twenty countries and territories that responded to the survey (as shown in Table S1) reported that their locality was ‘rabies-free’ or ‘non-endemic’ i.e. by the World Health Organization (WHO) criteria, no indigenous case of rabies (human or animal) was reported within the last two years in the presence of a robust surveillance system [35]. Only 20% of these non-endemic areas indicated that a control program (i.e., import control, vaccination, and surveillance) was implemented to achieve their non-endemic status.

At the time of survey administration, responses indicated that animal rabies cases were nationally notifiable in all rabies-endemic areas but in only 85% of non-endemic countries/ territories (see Table S1). Likewise, rabies in humans was nationally notifiable for all rabies-endemic cases, but in only 75% of non-endemic countries/territories.

### 3.2. Risk of Rabies Introduction

The risk of rabies introduction was considered low by the majority (60%) of non-endemic areas. However, St. Maarten and the British Virgin Islands deemed the risk to be high, whereas the remainder indicated intermediate-risk levels. Illegal importation of dogs from endemic areas was identified as a major risk for the introduction of rabies into almost all (95%) reporting non-endemic areas. For Trinidad, Guyana, and French Guiana, illegal importation of dogs and cats posed a risk for the re-introduction of canine-transmitted rabies, and wildlife migration was noted to be an additional risk with respect to sylvatic (bat-transmitted) rabies in Grenada, Trinidad, Suriname, and Belize.

### 3.3. Rabies Case Burden

#### 3.3.1. Animal Rabies

As illustrated in Table 1, in general amongst the rabies-endemic countries/territories, most animal cases (>20 cases per year) reported on the survey were from the Dominican Republic, Haiti, and Puerto Rico. Among countries where there was rabies surveillance, the fewest cases occurred in French Guiana (0–1 cases per year). No cases were reported from Suriname, where there was no rabies surveillance. In the other localities with vampire bat-transmitted rabies (i.e., Belize, French Guiana, Guyana, and Trinidad), the main animals affected were cattle. In Trinidad, small ruminant cases were also common. In other areas (Cuba, Grenada, and Puerto Rico), the majority of cases occurred in mongoose. Cats were also a notably affected species in Cuba, Haiti, and Grenada, with the latter also reporting cases in small ruminants transmitted by mongoose. Similarly, in Haiti and the Dominican Republic, where dog-transmitted rabies was prevalent, the majority of cases occurred in this species.

#### 3.3.2. Human Rabies

Table 2 illustrates the occurrence of human rabies cases within Caribbean rabies-endemic localities. Haiti had the highest human mortality with 6–10 cases estimated to be reported per year; however, sub-notification was suspected. Isolated cases were reported from French Guiana (2008), Puerto Rico (2015), and Suriname (1998). The Dominican Republic and Cuba reported cases more often, with the last reported cases being less than 5 years prior to this study. No cases were reported in Belize, Grenada, and Trinidad for at least 25 years. Guyana did not report the occurrence of any human cases in this survey. Dog and cat bites were reported to be the main source for human RABV exposure in Belize, Haiti, and Puerto Rico, and bat exposures were likewise reported in French Guiana and Cuba. In Grenada, mongoose exposures were listed as most common.

### 3.4. Rabies Case Definitions, Protocols, and Legislation

The existence of case definitions for suspect animal rabies cases was reported in most rabies-endemic localities (80%) but only in 20% of the non-endemic localities. Thirty percent (30%) of ‘rabies-free’ localities indicated that they had a case definition for a human rabies suspect, whereas 60% of rabies-endemic respondents had such a definition including those areas that had reported human cases. Only eight non-endemic localities (40%) reported having standard protocols in place for dealing with a suspected or confirmed animal rabies case compared to 70% of endemic areas. Likewise, 30% of non-endemic localities had protocols for dealing with a suspected or confirmed human rabies case compared to 60% of endemic rabies countries. Specific national legislation for the prevention and control of animal rabies existed in 70% of endemic and only 45% of non-endemic localities.

### 3.5. Rabies Virus Exposure Incidents

Human bite incidents from potential rabies animal reservoirs were reportable in 70% of endemic localities (Table S2), with less than half of these specific to bites from bats (French Guiana, Cuba, and Belize). Dog bites in humans were reportable in French Guiana, Cuba, Haiti, Belize, and Grenada. Animal bite incidents from potential rabies reservoirs were reportable in 70% of endemic localities, with Suriname, Belize, and Cuba being the exceptions. Incidents of humans being bitten by potential rabies virus reservoirs were reportable in 45% of non-endemic areas, with dog bites being the most common type of bite incident specified and Guadeloupe being the only respondent to specify the inclusion of bat bites. Only 30% of non-endemic areas indicated that animal bite cases from other animals considered potential rabies reservoirs were reportable, with dog bites being the most common and bat bites not specified for any country.

### 3.6. National Agencies with Responsibility for Rabies Surveillance

All endemic localities reported having governmental departments responsible for rabies surveillance in either animals or humans. In the Spanish-speaking Caribbean (Cuba, Dominican Republic, and Puerto Rico), the health ministry was responsible for both animal (canine) and human rabies surveillance, whereas in the other endemic localities, responsibilities for animal and human surveillance were held by the agriculture and health ministries, respectively. In non-endemic areas, 35% of respondents had departments responsible for syndromic surveillance for human rabies and 50% had departments responsible for animal rabies surveillance (passive). In contrast, in rabies-endemic localities, nearly all (90%) indicated that they had governmental departments responsible for human rabies surveillance, and all reported having departments responsible for animal surveillance (although no surveillance was noted to be conducted in Suriname).

### 3.7. Rabies Surveillance Programs in Animal Populations

#### 3.7.1. General

All endemic localities, apart from Suriname, indicated they conducted some form of surveillance for animal rabies. Passive surveillance was conducted in domestic and wildlife populations in all other (9) endemic areas, with testing of suspected cases for rabies virus antigens based on the reporting of clinical signs and human exposures. For example, in the Dominican Republic, an average of 2–3 (brain) samples from suspect animals was estimated to be tested per week. Active surveillance for rabies was less commonly implemented and mainly focused on rabies reservoirs in endemic areas. For the purpose of this study, active surveillance was defined as targeted sampling and testing (for antigen or antibody) of specific groups of animals based on risk factors.

#### 3.7.2. Wildlife

Routine active surveillance was conducted in the bat and mongoose populations in Trinidad and Puerto Rico, respectively. In Grenada, mongooses killed by animals or humans were opportunistically submitted for diagnostic testing of brain tissue. Likewise, in Guadeloupe, only bats that were involved in human bite incidents were submitted to the Institut Pasteur (Paris) for testing. In Haiti, a rabies prevalence research project was being conducted in the bat population at the time of administration of this survey and a similar project in the mongoose population was pending. Similarly, in Curacao, although the disease was not endemic, a research project was reported as being implemented to test for rabies within the bat population.

#### 3.7.3. Domestic Animals

Opportunistic (passive) rabies diagnostic testing of dogs and cats was conducted in 55% of non-endemic localities. This was based on the reporting of public health exposures and clinically suspect animals. Active surveillance in domestic animals was mainly conducted in rabies-endemic areas with meso-carnivore rabies. The stray dog population was the main target for this type of surveillance in Cuba, Haiti, and Grenada, with Cuba estimating a sample size of 0.1% of their dog population.

### 3.8. Animal Rabies Vaccination

As illustrated in Table 3, with the exception of Suriname, routine vaccination programmes for various animal species were conducted for all endemic areas. The vaccine was provided free of charge in six (60%) of the rabies-endemic countries (Belize, Cuba, Grenada, Haiti, Dominican Republic, and Trinidad). Four (40%) of the rabies-endemic localities indicated that they experienced unspecified difficulties in obtaining animal rabies vaccines for routine vaccination. The main target populations for animal vaccination strategies were herbivores (mainly cattle) and domestic carnivores (mainly dogs). Dogs were the main species vaccinated in Belize, Cuba, the Dominican Republic, Grenada, and Haiti, with the highest population coverage (>90%) estimated for Cuba. Ruminants were targeted in Guyana, French Guiana, and Trinidad, with Trinidad reporting the highest estimated population coverage (70%) and Guyana the lowest (10%). Rabies vaccination was generally a recommended practice in most of these areas, as compared to being legislatively mandatory in French Guiana and Trinidad.

### 3.9. Human Pre-Exposure and Post-Exposure Prophylaxis (PEP)

As shown in Table 2, pre-exposure rabies vaccination for at-risk personnel was routinely conducted in all endemic areas and included veterinarians, veterinary students, laboratory personnel, and animal health field assistants. Only 6 (30%) of non-endemic countries/territories conducted human vaccination for rabies. Immunized persons included bat researchers (Aruba and Curacao), travellers to high-risk areas (Bermuda), laboratory personnel (Guadeloupe and Martinique), and veterinarians and animal health field assistants (Turks and Caicos and Martinique). All 7 countries/territories (6 endemic, 1 non-endemic) that responded to the survey question indicated that they used modern cell culture vaccines for immunization. At the time of survey, rabies immune globulin for PEP was only available in 8 (27%) Caribbean localities, 5 (50%) endemic (as shown in Table 2), and 3 (15%) non-endemic (Bonaire, Bermuda, and Guadeloupe). All endemic countries also had vaccine available for PEP.

### 3.10. Animal Reservoir Population Control Programs

National programs to control rabies animal reservoir populations were present in 70% of rabies-endemic locations. Dog spay and neuter programs were existent for population control in 50% of endemic countries. Although no rabies cases occurred, 30% of non-endemic respondents indicated that they implemented dog and cat spay and neuter programs for animal population control. These were mainly implemented by non-governmental organizations in both endemic and non-endemic countries. In two countries where vampire bat rabies was endemic, anticoagulant poisoning of these bats was conducted for population control. Similarly, at the time of survey administration, culling was also noted to be conducted for the mongoose populations in two countries where mongoose rabies was endemic.

### 3.11. Rabies Laboratory Diagnostics

Only Cuba, the Dominican Republic, and Puerto Rico conducted human rabies diagnostic testing, but animal testing was carried out in these three countries, as well as in Grenada, Haiti, and Trinidad. The direct fluorescent antibody test (DFA) was available in all the laboratories detailed above with the capacity for rabies diagnostics, but PCR was only routinely available for rabies diagnosis in Cuba and Grenada. The direct rapid immunohistochemistry test (dRIT) and histological techniques for rabies diagnosis were also used in Haiti. Belize, Guyana, and Suriname indicated that they expected to set-up facilities for animal rabies diagnostics within the next 5 years, and Haiti was the only country that indicated plans to set up facilities for human rabies diagnostics. Laboratories external to the Caribbean region to which samples were sent for animal rabies diagnostics included the Ministerio de Desarrollo Agropecuario Laboratory in Panama, Laboratorio Nacional de Salud in Villa Nueva in Guatemala, the Animal Health and Veterinary Laboratories Agency (AHVLA) in the United Kingdom, the Institut Pasteur de Paris in France, and the Rabies Program at the Centers for Disease Control and Prevention (CDC) in the United States of America.

### 3.12. National Import Health Regulations Related to Rabies

National import health restrictions related to rabies were in place for almost all rabies non-endemic and endemic localities. These applied mainly to dogs, cats, and wild carnivores and essentially included a requirement for rabies vaccination with or without rabies titre testing. In instances in which titre testing was also required, the minimum time allowed between vaccination and sample collection was most commonly one month but ranged from 3 weeks to 3 months. Most rabies non-endemic (90%) and endemic (80%) localities indicated that they had protocols in place for dealing with imported animals that did not meet their import health requirements. These mainly included re-exportation or refusal of entry (73%), euthanasia (66%), and quarantine with or without rabies vaccination (30%). For 26% of all respondents, the course of action in implementing these protocols was reported to differ between importations from OIE listed rabies non-endemic and endemic countries. Nine (30%) (4 non-endemic and 5 rabies-endemic) localities reported having a quarantine station for animal importation. Species of animals that could be housed mainly included equine (77%), dogs and cats (66%), ruminants (66%), avian (11%), and wildlife (22%). The length of the quarantine period varied according to species, ranging from 7 days (equine) to 6 months (dogs and cats).

## 4. Discussion

### 4.1. Rabies Status and Reservoir Hosts

The current pattern of canine-transmitted rabies in the English Caribbean noted in this study may reflect the historical timing of control in Great Britain (1922) [36]. This early date of elimination, with similar disease control and animal movement control measures aided by sea borders, may explain the apparent absence of indigenous cases of canine rabies on British island colonies over the last century. Thus, although 80% of non-endemic localities reportedly had no specific measures aimed at achieving a ‘rabies-free’ status, regulations and policies instigated during colonial times might account for the historical absence of the disease. In contrast, vampire-bat rabies present since pre-Colombian times [9], as illustrated in this survey, is still enzootic in Caribbean countries from which it was reported, possibly due to increased awareness, after the Trinidadian epidemic in the 1930s, i.e., Guyana, Suriname, Belize, and French Guiana [15,18,19,20]. Although the common vampire bat (*Desmodus rotundus*) is the main bat species implicated in RABV transmission in the Caribbean [37,38,39,40], given the occurrence of cross-species transmission events and RABV isolations associated with non-hematophagous bats [22,41,42,43,44], surveillance of these species should also be considered. 

Mongoose rabies was first recognized in Puerto Rico in 1950 [45], but suspicious clinical signs have been observed in mongooses since the beginning of the 20th century [46]. The disease was subsequently confirmed in the mongoose populations of Grenada (1955) and Cuba (1956) [46,47], where they remain the primary reservoir, and in mongooses in the Dominican Republic (1950) [48], which only became a significant reservoir for transmission events in the 1980s [46]. Although this species was not noted as an animal reservoir in Haiti, given the shared land boundaries with the Dominican Republic, it is very likely to also exist in Haitian mongooses [46,48,49]. The small Indian mongoose (*Herpestes auropunctatus*) is not indigenous to the Caribbean and was introduced from India to Guyana, Suriname, French Guiana, and 29 Caribbean islands in the late 19th century in an attempt to control rodents in colonial sugar cane plantations [50,51,52]. Phylogenetic studies of mongoose RABV variants in Cuba, Grenada, and Puerto Rico indicate that they are distinct meso-carnivore RABV variants, independently derived from dog-maintained viruses through separate introductions [9,42,53,54]. Therefore, the restricted distribution of mongoose rabies despite the extensive distribution of the vector within the Caribbean may be due to the historical absence of endemic RABV in the dog populations and lower mongoose population densities in most of the Caribbean localities [49] that did not facilitate either an initiating viral spill-over event or sustain effective animal-animal transmission rates. Other carnivore species thought to be involved in sylvatic rabies in the Caribbean include the gray fox (*Urocyon cinereoargenteus*) in Belize.

### 4.2. Risk of Introduction

The perception by the majority of non-endemic respondents that illegal importation is a major risk for canine-rabies introduction is justifiable given the scale of the Caribbean tourism industry. The high movement of humans and companion animals both from external regions and from island to island may facilitate the entry and rapid spread of canine-transmitted RABV, as the dog population is largely unvaccinated. Animal migration as a risk for the introduction or re-introduction of rabies viruses is most pertinent to countries such as Guyana, Suriname, French Guiana, and Belize that share land borders with other countries in which enzootic canine rabies still exists. For example, in 1995, after a 5-year absence of urban rabies, a limited outbreak occurred in Belize [55], which may have been attributed to animal movement from a neighbouring country. In other countries, such as Cuba and Grenada, bat migration has proven to be a threat. The identification of a vampire RABV variant and other RABV isolations in non-hematophagous bats in Cuba (see Table S3) [42,56], along with the presence of high RABV neutralizing antibody in *Artibeus* bats in Grenada [57,58], indicates that bat rabies may not be limited by the geographical range of the vampire bat, and the risk of bat RABV introduction may have been underestimated. The wide distribution of bats with high dispersal ability such *Tadarida brasiliensis* and *Molossus molossus* [59] may be associated with an increased risk of RABV translocation from bat rabies-endemic to non-endemic areas. This risk is highest for Caribbean countries in close geographical association with those countries in which bat rabies exists (e.g. Cuba in the north and Grenada in the south). The importance of RABV variant typing to identify reservoir bat species and elucidate possible origins cannot therefore be overemphasised. 

### 4.3. Surveillance and Reporting of Animal Rabies

In general, areas in which canine rabies still predominates are responsible for most of the animal rabies cases (mainly dogs and mongoose) in the Caribbean, which may be because dogs are more common aggressor species. Haiti accounts for the majority of dog cases, with consistently high annual estimates of animal cases despite gross under-reporting due to inconsistent surveillance (Table 1). For example, the implementation of a novel, community-based surveillance system from 2013–2014 led to the confirmation of 70 rabid cases from only 20% of Haitian communes, compared to 12 cases reported countrywide for the previous 4-year period using existing surveillance methods [33,60]. This clearly demonstrates the usefulness of implementing novel surveillance techniques based on field evidence and/or statistical modelling in this country and can be extended to other countries. 

In the Dominican Republic, during the 1960–1970s, dogs accounted for most (94%) of the animal rabies cases [46,61]. However, in the 1980s, following the implementation of dog vaccination programs, a relative increase in rabid mongoose highlighted the importance of this introduced species in transmission [46], although as this study illustrates the dog remains the primary RABV reservoir and transmitter. A similar increase in mongoose rabies was observed in Cuba during this time, but in this case the mongoose displaced dogs as the main reservoir host in 1982 [41,46]. Wildlife rabies was increasingly diagnosed in Cuba thereafter with >17 bat rabies cases reported from 1991–1999 and current positivity rates up to 64% estimated for mongoose [42,46,56]. In Puerto Rico, since the detection of mongoose rabies in 1950, all rabies cases have been attributed to this viral variant [46,62,63], with mongooses accounting for about 75% of total animal rabies cases [64] and is shown to be the main animal affected in this study. Similarly, in Grenada, 73% of animal rabies cases reported during a passive surveillance program implemented by the Grenadian Ministry of Health for nearly two decades were mongoose [61,65]. The results of the current survey indicate that Cuba conducts routine active surveillance for dogs with an estimated target of 0.1% population coverage This activity should be more than adequate to identify circulation of endemic RABV given the minimum recommended sample size for effective surveillance is 0.01–0.02% of the dog population [21]. 

Of those localities in which the vampire bat is the main reservoir, Trinidad reported the highest estimated number of animal cases per year (6–10 cases). This may be due to a higher prevalence of cases and/or more efficient surveillance programs. Although there is evidence of RABV importation from the South American mainland to Trinidad [40], its smaller land mass and more manageable sea borders are advantageous in terms of disease surveillance efforts and case finding in comparison to continental areas. Additionally, the long history of rabies in Trinidad may have resulted in increased awareness and case reporting by the general public. Alternatively, the smaller size of the island means that forested areas are more accessible and the risk of bat contact with susceptible domestic animals and humans may be higher. In contrast, in mainland countries such as Suriname, cases occurring in the interior forested areas are likely to go undetected, especially given the absence of structured surveillance programs. In Guyana and French Guiana, where passive surveillance was conducted, Guyana reported a higher average case number (n = 1–5) than French Guiana (0–1) possibly due to the absence of routine animal vaccination programs and the use of clinical (non-laboratory) diagnosis of cases in Guyana. Clinical diagnosis may result in misdiagnoses with higher rates of case identification and should therefore be epidemiologically linked to at least one laboratory confirmed case.

Centralized reporting of national rabies cases through the use of regional data collection tools such as the online platform SIRVERA facilitates transparency and fosters trust between Caribbean countries [29]. However, data contribution is largely voluntarily, and few Caribbean countries have reported consistently [30]. Reporting should therefore be promoted at the national level to increase the availability of Caribbean data, and data quality checks should be implemented to improve the validity of data [29]. Increased participation of Caribbean nations and representation of the CaribVET network in the REDIPRA meetings in recent years will serve well to fortify consistency in reporting and encourage presentation of valid and accurate data. 

Routine or project-based rabies surveillance of bat populations has been conducted in at least eight Caribbean localities, with RABV isolated from 16 bat species (12 genera) in six localities (Table S3) [39,42,43,44,46,47,56,58,66,67]. While vampire bats are implicated in the majority of reports on bat-transmitted rabies within the Caribbean, and the vampire bat-related RABV variant has been the major variant detected, isolations in other bat species indicate they should be included in routine surveillance activities. The number of rabid species identified in each of the localities mentioned above is relatively low compared to their bat species richness [67,68,69,70,71,72,73,74,75] (Table S3). However, the number of rabid bat species is directly correlated to research effort [22], so it is anticipated that increased surveillance (and the use of modern laboratory techniques) will result in the identification of more rabid bat species. Since RABV isolations from Caribbean bats are rare, with recent detection rates <0.5% [39,40,76], passive surveillance with the testing of sick or dying bats for RABV and seroprevalence studies for both bat endemic and non-endemic locations would be preferable for initial studies in resource-limited settings. More expensive enhanced surveillance should then target bats species with higher dispersal abilities [59,77], those previously found to be rabies positive, and other species to which they have sympatric roosting relationships. Likewise, RABV variant diversity studies can focus on bat genera noted elsewhere in the Americas to host several variants (e.g. *Myotis*, *Eptesicus, Lasiurus, Tadarida,* etc.) [32]. Beyond bats that expose humans and domestic animals, or bats that appear ill or are found dead, indiscriminate killing of bats is unjustified, and non-destructive sampling is advocated.

### 4.4. Surveillance and Reporting of Human Rabies

In the Caribbean, human rabies surveillance relies on the expectation that cases will be reported if they occur. However, in the absence of a well-defined case definition (the basic foundation for disease surveillance programs), as was noted for some countries in this study, under-reporting and/or misdiagnosis are to be expected. Standardized case definitions provided by international bodies (e.g., OIE/WHO standard guidelines) should be adopted for national surveillance [35]. In theory, human rabies clinical surveillance falls under the auspices of the Ministry of Health in all endemic localities in the Caribbean. However, in both non-endemic and endemic locations where the last reported human case was not recent, i.e., Trinidad (1937) [78], Grenada (1970) [61], Belize (1989) [79], Suriname (1998) [24], and Guyana (2001) [80], surveillance mechanisms and laboratory capacities may not be readily available to support such activities. On the other hand, in places reporting human cases within recent years (i.e. Haiti, French Guiana), there may be other challenges such as incomplete reporting and inefficient data sharing between authorities handling human versus animal surveillance. Although no surveillance is being conducted in Suriname at present, an anecdotal trend of human rabies at 15–20 year intervals was reported in this survey, with outbreaks generally in the forested interior affecting an average of about 10 persons [20,24]. Epidemiological data such as this can be used to plan intermittent active surveillance activities to support case finding and public health management of exposures. In planning such activities, authorities should bear in mind that given the coastal population settlement structure in Suriname (which is similar to Guyana and French Guiana), urban surveillance systems may not capture human cases in the interior, especially in indigenous communities.

Overall, the results of the present survey indicate that localities where canine-transmitted rabies predominated had higher numbers of human rabies cases. This may be due to the close social relationships between humans and dogs, and their greater accessibility to human populations, especially in light of free-roaming dog-keeping practices [81]. Official reports of canine-transmitted human rabies in the Americas are generally recognised as underestimates. Therefore, disease modelling has been employed to estimate a more realistic number of about 200 human cases per year [35]. Haiti, which reported only 2 to 39 cases per year from 1970 to 2013 [24,82,83] and 6-10 cases per year in the present study, was predicted to account for the majority of these [35]. Apart from under-reporting, Haitian rabies case numbers are thought to be highly variable and possibly correlated to the prevailing environmental conditions, such that increased cases have been observed after natural disasters, e.g., in 2006 (11 cases) after a series of hurricanes and in 2010 (13 cases) after a major earthquake [23,25,32]. This observation may be a result of post-disaster challenges to the national dog rabies vaccination campaign due to competing needs and increases in the stray dog population. These challenges should therefore be anticipated and budgeted for when establishing post-disaster management plans.

Although the Dominican Republic shares the island Hispaniola with Haiti, human rabies mortality rates are considerably lower and are likely correlated to the disparities in socioeconomic conditions. As in Haiti, the dog is the major reservoir for rabies and is involved in most transmissions. However, with the decline in canine cases [84] and recognition of mongoose as a vector [46], the prevalence of human cases in the Dominican Republic gradually decreased from an average of 5 cases annually during the 1980s [46] to sporadic cases every 1–2 years as reported here. A similar trend in human cases was seen in Cuba [24,84], which is largely attributed to the reduction in canine-transmitted rabies due to the PAHO regional program [25]. The last reported human case of canine-transmitted rabies in Cuba was in 2008 [30]. Wildlife-transmitted human rabies emerged in 1988 with an increase in human cases associated with non-hematophagous bats, following the first occurrence in 1970 linked to an *Eptesicus fuscus* bat [30,41,42]. Currently, human rabies cases average less than one per year in Cuba, and are transmitted by wildlife (mongoose and bats) and cats, with the last case documented in 2016 [30,85]. On the other hand, in Puerto Rico, the last human cases of canine-transmitted rabies (canine RABV variant) occurred relatively early in 1896, with the later elimination of the disease in 1933 [45,47]. Since the establishment of mongoose rabies in Puerto Rico [45], few human rabies cases have been reported despite high numbers of animal cases. The last confirmed human rabies case in Puerto Rico was in 2015, due to a mongoose bite [86].

In areas such as Haiti and the Dominican Republic, in which under-reporting of human cases is thought to be high, active surveillance mechanisms employing community-based case-searching may be used to attain a more accurate picture of the disease burden. This can in turn be used as evidence to leverage political commitment and initiate policy development for disease prevention and control. Furthermore, in an attempt to evaluate the sensitivity of surveillance mechanisms to identify human rabies cases, systemic evaluations of surveillance outcomes, for example, and reviews of medical records for acute encephalitis, can be conducted [27]. In localities where there is separation of responsibilities for animal and human rabies surveillance, inter-sectoral coordination is essential to avoid inefficiencies. In addition, in those countries with shared land borders, inter-administrative and departmental cooperation is crucial to control efforts.

### 4.5. Human Exposures and Public Health Risk

Public health risk varies according to the type of animal reservoir and nature of animal–human interaction. In general, places with canine-transmitted rabies will have a higher risk than those with wildlife rabies, because carnivore reservoirs (e.g., mongoose, foxes) have a higher potential for human contact than other reservoirs such as bats. Nevertheless, in the Caribbean, bat bites are generally overlooked as potential RABV exposures, particularly in countries without vampire bats. However, given evidence of routine bat movement between islands and RABV isolations from countries without vampires, the risk of transmission from such incidents should not be underestimated [42,57]. In Trinidad, despite reports of bat biting to dogs, no cases of rabies in domestic carnivores have been documented since the 1930s [76]. Conversely, French Guiana had several confirmed domestic carnivore cases associated with the vampire bat RABV variant within the last 30 years [87], most recently in 2015 when more than 10 people were exposed to an infected dog [88]. This demonstrates that the presence of canine variant RABV is not a prerequisite for transmission by dogs. This risk is recognized in French Guiana, where dog bites are reportable, but this is not the case in Trinidad, which increases the probability of unrecognized human exposures on the island.

### 4.6. Rabies Diagnostic Capacity

Due to the ubiquity of vampire bat rabies and previous high prevalence of canine-transmitted rabies in Latin America, there are extensive rabies laboratory networks throughout this region [89]. In contrast, the wide disparities in the rabies situation within the Caribbean have not supported such a system, with most islands having no internal capacity for rabies testing. The only Caribbean localities noted by the present survey to have diagnostic capacity for human rabies were the Spanish-speaking Caribbean countries, which, similarly to their Latin American counterparts, have one primary agency responsible for rabies surveillance in both human and animal populations. At present, given where human cases routinely occur, these facilities are ideally positioned for efficient human rabies diagnosis and regional control, although in some cases this will only be realised if there are mechanisms for country to country cooperation. For example, Haiti, which is responsible for the vast majority of human cases, has no dedicated human rabies diagnostics facility but shares the island of Hispaniola with the Dominican Republic, where facilities exist.

Our study shows that most endemic localities currently have laboratory capacity to conduct DFA testing for animal samples, including a recently-established animal rabies laboratory in Guyana. However, in the majority of these countries, limitations related to dedicated funding for reagents and consumables may hinder consistent testing. In such cases, use of alternative, low-resource diagnostic tools (e.g., immunohistochemical and immunochromatographic techniques) [90] and the pooling of samples (especially for wildlife) may be of immense value. In 2014, CaribVET organized a training workshop on rabies diagnostic techniques for rabies-endemic Caribbean countries. Such training exercises, together with strong laboratory networking facilitated by CaribVET, can significantly improve rabies diagnostic capacities in countries without existing laboratory facilities. At present, only two Caribbean localities routinely employ molecular diagnostics for rabies. It may be useful to develop this capacity in other countries or to outsource testing regionally to WHO/OIE reference centers for confirmatory testing, especially with public health exposures, and for viral typing, which can be useful towards determination of RABV variants and reservoir host identification.

### 4.7. Prevention and Control Measures

#### 4.7.1. Animal Vaccination

Animal rabies vaccination is targeted at those animals identified as high-risk species based on the behaviour of the main reservoir hosts, so in endemic Caribbean areas the main targets are domestic carnivores and herbivores. In the face of limited financial resources, national programs that attempt mass vaccination schemes annually (see Table 3) may achieve lower population coverage compared to those with biennial and triennial schemes. Economic constraints may be off-set by vaccine procurement mechanisms initiated by international bodies, such as the PAHO revolving fund (canine vaccine) [91] and the OIE vaccine bank [92]. However, some localities may still be limited in the availability of personnel to effectively administer regular national vaccine programmes. For example, despite legislative support for effecting mandatory animal rabies vaccination programmes in Trinidad and French Guiana, manpower to enforce and conduct programmes is a significant limiting variable [87,93].

In areas with endemic canine rabies, vaccine coverages of at least 70% in the dog population over a few years should be effective to prevent the enzootic circulation of RABV [35,94]. Given an estimated vaccine coverage of less than 50% in Haiti, elimination of canine-transmitted rabies by 2030 [95] may prove to be less realistic than in places such as the Dominican Republic and Cuba with reported coverages over 80%. Although the results of this survey indicate that Cuba has the highest estimated dog population vaccine coverage in the Caribbean, this figure does not include free-ranging dogs, so the at-risk canine population is underestimated. This likely also holds true for the estimates of canine vaccine population coverage in other endemic areas. In non-endemic areas where routine vaccination programs do not occur, rabies vaccine coverage of dog populations would be low, and introduction of RABV to these naïve populations could result in explosive outbreaks. With the exception of Haiti, areas with carnivore rabies historically implemented major dog vaccination programs, e.g., Grenada (1971), Cuba (1981), and the Dominican Republic (1987), which resulted in effective declines in endemic dog rabies over successive years [46,61]. Current estimates of rabies immunization coverage for the Grenadian dog population given in other studies (20–25% of the dog population) [54,96] are much more conservative than those provided in this survey. Similarly, in Haiti, dog vaccination coverages reported elsewhere were 40–42%, compared to 50% in this study [83,97]. Annual variations in vaccination coverage related to variations in the availability of funding may explain these inconsistencies. The absence of reliable animal population census information is an additional barrier to obtaining accurate vaccine coverage estimate in Caribbean animal populations.

In most places with endemic vampire bat-transmitted rabies, routine rabies vaccination is conducted in livestock, with particular emphasis on cattle. Compliance with such rabies vaccination programs is influenced by whether there is a legal requirement and the cost to the farmer. For example, in French Guiana, one study showed a 7% decline in livestock vaccination from the biennial periods 2006-2007 (when vaccine was free of cost) to 2008–2009 after a ministerial decree (2008) that implemented shared costs between livestock farmers and the government for mandatory vaccination [87]. Although no population coverage data were provided in the current study, one report indicated that 41% of livestock (including 26% small ruminants) were vaccinated during the period 2008–2009 in French Guiana [87]. As noted in the current study, vaccination coverage for livestock varies widely between countries, with differences attributed to the type of rabies immunization program implemented. The first mass livestock rabies inoculation program in the Caribbean was implemented in Trinidad during 1932 [98] and has since been conducted on a routine basis for rabies prevention. On the other hand, livestock rabies vaccination in Guyana is conducted irregularly on a more concentrated scale, as ring-vaccination schemes, after clinical cases are observed. Ring-vaccination around rabies index cases are also implemented in Trinidad, although the radius range around cases may need to be increased to coincide with the vector feeding range [93].

#### 4.7.2. Human Pre-Exposure and Post-Exposure Prophylaxis (PEP)

Pre-exposure human rabies vaccination was conducted in all of the rabies-endemic areas; however, the results of this study did not indicate how and when immunity was monitored in these individuals. Although the WHO recommends routine rabies titre determination for laboratory workers and animal health professionals [35], in the Caribbean this is difficult to maintain, given limited resources and no laboratory capacity for rabies serological testing. Therefore, most countries administered booster vaccines based on duration of immunity estimations for the vaccine without titre testing to determine the necessity of booster vaccines (i.e., titer levels <0.5 IU/mL [35]). This system, however, does not take into account the possibility of seroconversion failures and subsequent lack of immunity. The importance of pre-exposure vaccination for visitors to Caribbean rabies-endemic areas involved in potentially high-risk activities is highlighted by reports of imported human cases into other localities to which the Caribbean was epidemiologically linked as the source. These cases are mainly from Haiti, with four cases exported to the United States of America and the Netherlands during the period 1994–2013 [82]. The neighbouring Dominican Republic was also implicated in a human case diagnosed in Canada in 2012 [99].

In Latin America, despite the significant decline in human rabies cases over the last few decades, the demand for rabies vaccine has increased [27]. This may have been due to increased disease awareness driving greater adherence to prophylaxis protocols after exposures. In the Caribbean, similar increases may be expected with the implementation of rabies awareness programs, particularly in endemic areas during domestic animal outbreaks. In contrast, in localities where rabies cases were not reported for many years, institutional memory may fade and cause an underestimation of the risk of RABV transmission, which would promote non-compliance to prevention and control strategies. As Haiti bears the heaviest burden of human rabies cases in the region, the allocation of rabies vaccine is much higher than regional counterparts, with approximately 8,000 doses of human vaccine administered per year [83]. While only 7 (23%) respondents answered the question, cell-culture rabies vaccines were noted to be used by all of these countries, including the Dominican Republic, where nerve tissue vaccines were only discontinued in 2009 [35]. Provisions made by the PAHO revolving fund [91] have made modern cell culture human rabies vaccines more accessible and affordable. The poor response to survey questions related to human rabies prophylaxis may be a result of the survey having been primarily administered to veterinary authorities that may have limited the data captured from the human health sector, especially in areas where separate institutions are responsible for human and animal rabies. The limited availability of rabies-immune globulin (RIG) throughout the Caribbean noted in this study can pose significant challenges to effective PEP in humans. Bat bite incidents in unvaccinated humans are considered category III (severe) exposures warranting both vaccine and RIG [35]. However, at present, 80% of bat rabies-endemic countries do not have the biologic available locally, which puts the population at risk in the event of bat exposures. To bridge this gap in data and to ascertain the current knowledge of human medical practitioners on rabies in general, a similar study targeting professionals in the field of human medicine is currently on the workplan of the CaribVET Rabies Subgroup to be conducted within the Caribbean.

#### 4.7.3. Reservoir Host Population Control Programs

Reservoir host population control mainly involves culling and reproductive sterilization techniques. For domestic mammals, dog and cat spay and neuter programs were the most common population control measures reported across the Caribbean, including in non-endemic localities, and were closely associated with national stray dog management programs. While vaccination effectively curbs enzootic circulation of canine-transmitted RABV [32], population control programs are useful to reduce the stray animal population, which can form pockets of unvaccinated animals and a nidus of enzootic virus. In wildlife, population control programs were based on chemical culling methods, e.g., anticoagulant pasting of vampire bats. Trinidad has a long history of studies on chemical control for vampire bats, with initial programs resulting in significant reductions in estimated bat numbers [93]. The island, due to its relative insular isolation compared to its mainland counterparts, can facilitate studies on other methods of rabies management similarly based on the ecology of the vampire bat, such as topical oral vaccination schemes [100]. Mongoose control programs identified in this study mainly involved culling by poisoned baiting; however, the potential of chemical sterilization and oral vaccination of this species are now being investigated in pilot programs [101]. These strategies should provide more ecologically sound and sustainable population control programs for rabies animal reservoirs.

## 5. Conclusions

Rabies epidemiology across the Caribbean differs from the situation in Latin America and North America. Historically, wildlife rabies in the Western Hemisphere was first suspected in this region. However, with the possible exception of Trinidad, programs for control of wildlife rabies are not as well established or consistent as those in these other regions, mainly due to financial constraints. The diversity and inconsistencies between rabies programs within the Caribbean make it difficult to establish a standard regional approach to rabies control and prevention, as was done in Latin America. More realistically, best practice guidelines for rabies control independently addressing the various RABV reservoirs can be developed based on evidence-based information and adapted to the individual epidemiological status, existing infrastructure, and available resources in the implementing country. The mongoose is one of the main rabies reservoirs in the Caribbean, but due to its absence from other regions in the Americas, it is often overlooked as a transmitting animal, which can lead to fatal consequences [102].

Sustained infection of dog-transmitted rabies in areas such as Haiti, where severe economic challenges exist, presents barriers to the elimination of the disease in the region. If the goal of elimination is to be attained in such areas, consistent external support will be necessary. As noted in other regions, since short-term goals are more suited for encouraging donor investment [27], it might be worthwhile implementing programs in phases. A minimum requirement for the certification of a country as being ‘rabies-free’ is a functional laboratory-based surveillance program. However, rabies diagnostic capacity is limited within the Caribbean, which may compromise surveillance efforts and pose challenges to national declarations of rabies freedom. Training and laboratory networking such as that facilitated by CaribVET can function to improve rabies diagnostic capacities and surveillance in Caribbean countries. Furthermore, even if the elimination of canine rabies is on the distant horizon for the Caribbean, the approach to wildlife rabies control, related to mongoose and particularly bat-transmitted rabies, is not as straightforward, and the possibility of realistic prevention and control in these populations is debatable. The latter may be a more long-term goal with the adoption of measures successfully utilized in developed countries, e.g., oral vaccination programs [103] with the application of novel species appropriate techniques, such as vaccination of mongoose populations and perhaps vampire bats by oral administration during routine feeding and social grooming [100]. Nevertheless, coordinated efforts towards improving rabies control in the Caribbean should be continued through networks like CaribVET, which represents an asset to the region by promoting communication, increasing awareness, developing strategies, and sharing of human and material resources between Caribbean countries.

## Figures and Tables

**Table 1:** Rabies endemic locations in the Caribbean: main animal reservoir and average case frequency with types of animals affected by country.

Location	Main Animal Reservoir	Mean # Cases per Year	Main Animals Affected
Belize	Vampire bat	1–5	Cattle
Cuba	Mongoose	16–20	Dogs, cats, mongoose
Dominican Republic	Dog	>20	n.r.
French Guiana	Vampire bat	0–1	Cattle, dogs, bats
Grenada	Mongoose	1–5	Mongoose, cattle, small ruminants, dogs, cats
Guyana	Vampire bat	1–5	Cattle
Haiti	Dog	>20	Dogs, cats
Puerto Rico	Mongoose	>20	Mongoose
Suriname	Vampire bat	0 ^a^	Bats ^a,b^
Trinidad	Vampire bat	6–10	Cattle, small ruminants

^a^ no surveillance; ^b^ not laboratory confirmed; n.r. (no response).

**Table 2:** Human rabies cases in the Caribbean: case frequency and availability of human rabies biologics for pre-and post-exposure prophylaxis reported in the survey.

Location	Years since Last Reported Case *	Mean ^#^ Human Cases per Year *	Causative Variant of Last Human Case	Pre-Exposure Vaccination	Biologics Available for Post-Exposure Prophylaxis
Belize	>20	0	Canine	Yes	vaccine
Cuba	1–5	0–1	Mongoose	Yes	vaccine and RIG
Dominican Republic	<1	0–1	Canine	Yes	vaccine
French Guiana	6–10	0–1	Bat	Yes	vaccine and RIG
Grenada	>20	0	Mongoose	Yes	vaccine and RIG
Guyana	n.c.r.	0	n.c.r.	Yes	vaccine
Haiti	<1	6–10 ^#^	Canine	Yes	vaccine and RIG
Puerto Rico	1–5	0–1	Mongoose	Yes	vaccine and RIG
Suriname	11–20	0–1	Bat	Yes	vaccine
Trinidad	>20	0	Bat	Yes	vaccine

* as at June 2018; ^#^ sub-notification suspected; n.c.r.: no cases reported on survey; RIG: rabies immune globulin.

**Table 3:** National animal rabies vaccination programs.

Location	Vaccination Requirement	Main Animals Vaccinated	Periodicity	Estimated Population Coverage
Belize	recommended ^##^	Dogs and cats	annually	80% dogs
Cuba	recommended ^##^	Dogs	annually	>90% dogs ^¶^
Dominican Republic	recommended	Dogs and cats	n.r.	80% dogs and cats
French Guiana	mandatory by legislation	Ruminants, dogs and cats, equine	biennially	No data available
Grenada	recommended ^##^	Ruminants, dogs and cats, equine	annually	40% dogs ***; 4% cats; 31% goats; 22% sheep; 4% cattle
Guyana	recommended	Bovine	annually	10% bovine
Haiti	recommended ^##^	Dogs	annually	40—50% dogs
Puerto Rico	recommended	Dogs and cats, ruminants, equine	annually	No data available
Suriname	Not conducted	-	-	-
Trinidad	mandatory by legislation ^##^	Ruminants, equine	triennial	70% bovine; <40% goat and sheep; <40% equine

^¶^ owned dog population (not inclusive of stray population); ^##^ vaccination provided free of charge; *** not inclusive of vaccination done by private (non-government) veterinarians; n.r.: no response.

## Supplementary Materials

The following are available online at http://www.mdpi.com/2414-6366/3/3/89/s1, Table S1: Rabies disease status and national notification status of Caribbean countries/ territories surveyed; Table S2: Mandatory reporting of rabies virus exposure incidents in humans and animals and: Table S3: Bat species richness and species identified as rabies positive in the Caribbean. Questionnaire used for the Caribbean Animal Health Network (CaribVET) regional rabies survey administered in this study.
