# Supplementary material for: Rabies in the Caribbean: A Situational Analysis and Historic Review
Source: Trop Med Infect Dis. 2018 Aug 20;3(3):89. doi: 10.3390/tropicalmed3030089 (PMC6160905; doi:10.3390/tropicalmed3030089)
Supplement: Supplementary file 1 [file tropicalmed-03-00089-s001.zip › tropicalmed-343539-supplementary/Appendix A_CaribVet Regional Rabies Questionnaire.pdf]

CaribVet Veterinary Public Health Working Group

Regional Rabies Questionnaire

CONTACT DETAILS

Name

(First Name) **(Surname)**

Organization/ Ministry **Job Position**

Country

Telephone: **Fax:**

E-mail

COUNTRY STATUS

1) (a) What is the rabies status of your country?

☐ Endemic in domestic animals
☐ Endemic in wildlife (including bats)
☐ Sporadic cases in domestic animals
☐ Sporadic cases in humans
☐ Rabies free

(b) If rabies cases occur, what is the main animal reservoir of rabies in your country?

☐ Dog
☐ Bat
☐ Mongoose
☐ Other

(c) (i) If animal rabies free, did you implement a control program to become rabies free?

(ii) If yes, what did this entail?

(d) If animal rabies free, how would you classify the risk of rabies introduction into your country?

☐ High-risk
☐ Intermediate-risk
☐ Low-risk
☐ No-risk

(e) (i) What method(s), of animal rabies introduction is (are) most relevant to your country?

☐ Legal importation
☐ Illegal importation
☐ Other

(ii) Specify from which country/region:

(iii) Specify from what animal species:

2) (a) Is animal rabies a nationally notifiable (reportable) disease in your country?

(b) If yes, when was the last reported case of animal rabies in your country? estimate

- ☐ less than one year
- ☐ 1 - 5 years
- ☐ 6-10 years
- ☐ over 10 years
- ☐ or specify

3) (a) Is human rabies a nationally notifiable (reportable) disease in your country?

(b) If yes, when was the last reported case of human rabies in your country? estimate

- ☐ less than one year
- ☐ 1 - 10 years
- ☐ 11 - 20 years
- ☐ over 20 years
- ☐ or specify

4) (a) What is the average number of reported animal rabies cases per year? estimate

- ☐ 1 - 5 cases
- ☐ 6 - 10 cases
- ☐ 11 - 15 cases
- ☐ 16 - 20 cases
- ☐ more than 20 cases
- ☐ or specify ______

(b) What is (are) the main animal species affected?

- ☐ Cattle
- ☐ Small Ruminants
- ☐ Dogs
- ☐ Cats
- ☐ Bats
- ☐ Mongoose
- ☐ Other ______

5) (a) What is the average number of reported human rabies cases per year? estimate

- ☐ 1 - 5 cases
- ☐ 6 - 10 cases
- ☐ more than 10 cases
- ☐ or specify ______

(b) (i) If human cases occur, what is the main origin of these cases?

- ☐ Importation
- ☐ Resident animal bite/ scratch
- ☐ Other ______

(ii) If resident animal bite/ scratch, specify the animal species involved:

- ☐ Dogs
- ☐ Cats
- ☐ Bats
- ☐ Mongoose
- ☐ Ruminants
- ☐ Other

PROTOCOL AND LEGISLATION

6) (a) Do you have a case definition for an animal rabies suspect?

(b) If yes, please state what this is:

7) (a) Do you have a case definition for a human rabies suspect?

(b) If yes, please state what this is:

8) (a) Do you have protocols in place for dealing with animal suspect/ confirmed cases?

(b) If yes, please state what these are:

9) (a) Do you have protocols in place for dealing with human suspect/ confirmed cases?

(b) If yes, please state what these are:

10) (a) Are human cases of biting from potential rabies vectors (e.g. dogs, mongoose, bats) reportable in your country?

(b) If yes, which vector(s) is/ are applicable?

- ☐ Dog
- ☐ Bat
- ☐ Mongoose
- ☐ Other

11) (a) Are animal cases of biting from potential rabies vectors (e.g. dogs, mongoose, bats) reportable in your country?

(b) If yes, which vector(s) is/ are applicable?

☐ Dog

☐ Bat

☐ Mongoose

☐ Other

12) (a) Is there specific national legislation for animal rabies control, rabies case reporting or rabies treatment in your country?

(b) If yes, please indicate relevant legislation:

SURVEILLANCE

13) (a) Is there a national agency responsible for conducting human rabies surveillance in your country?

(b) If yes, please indicate relevant institution(s)/ agency(ies):

14) (a) Is there a national agency responsible for conducting animal rabies surveillance in your country?

(b) If yes, please indicate relevant institution(s)/ agency(ies):

15) (a) Do you have an active surveillance program for rabies in domestic animal species?

(b) If yes, for which animal species?

(c) If yes, how is surveillance conducted?

16) (a) Do you have an active surveillance program for rabies in wildlife?

(b) If yes, for which animal species?

(c) If yes, how is surveillance conducted?

17) (a) Do you have a passive/ enhanced passive surveillance program for rabies in wildlife and or domestic animal species?

(b) If yes, for which animal species?

(c) If yes, how is surveillance conducted?

RABIES VACCINATION AND BIOLOGICALS

18) (a) Does your country implement a national animal rabies vaccination campaign?

(b) (i) If yes, please indicated if it is:

- ☐ mandatory (legal requirement)
- ☐ recommended

(ii) If yes, what is the periodicity?

☐ Annually
☐ Biennially
☐ Every three years
☐ Other

(iii) If yes, what animal species are involved?

☐ Bovine
☐ Small Ruminants
☐ Canine
☐ Feline
☐ Equine
☐ Other

(iv) If yes, what is the estimated vaccine coverage (percentage) per animal species population (e.g. 70% of canine population; 100% cattle population):

v) If yes, is vaccination provided free of charge to animal owners?

☐ Yes
☐ No
☐ Not sure

19) (a) Do you experience any difficulties in obtaining animal rabies vaccines?

(b) Please indicate which vaccine formulation is used (type, brand and manufacturer):

(c) Is this an OIE approved vaccine?

(d) Please indicate vaccine supplier/ distributor (local agent):

20) (a) Do you conduct rabies vaccination for humans at risk (pre-exposure prophylaxis)?

(b) If yes, who are vaccinated?

- ☐ Laboratory personnel
- ☐ Field animal health personnel
- ☐ Veterinarians
- ☐ Other

(c) If yes, please indicate which vaccine formulation is used (type, brand and manufacturer):

21) (a) Does your country have a ready supply of biologics for rabies post exposure treatment in humans?

(b) If yes, which biologics are available?

- ☐ Vaccine
- ☐ Immunoglobulin
- ☐ Not sure

VECTOR CONTROL

22) (a) Does your country implement a national program to control and reduce the animal populations for rabies vector species?

(b) If yes, which animal species are targeted?

- ☐ Dog
- ☐ Bat
- ☐ Mongoose
- ☐ Other

(c) (i) What does this program involve?

- ☐ Spay and neuter
- ☐ Culling
- ☐ Chemical control
- ☐ Other

(ii) If culling is implemented, what is the method used?

(iii) If chemical control is implemented what chemical is used and how is it administered?

RABIES DIAGNOSTICS

23) (a) Do you conduct human rabies diagnostic testing in your country?

(b) If yes, which test(s) do you implement?

- ☐ DFA
- ☐ PCR
- ☐ Histopathology
- ☐ Mouse inoculation
- ☐ Serology
- ☐ Other

(c) If yes, do you receive human samples from other countries for testing?

(d) (i) If no, do you send human samples to laboratories in other countries for testing?

(ii) Which laboratories (including country) do you send human samples to for testing? (indicate type of samples)

(e) (i) If no, does your country have any immediate plans to set up rabies diagnostic testing for humans?

(ii) If your country plans to set up rabies diagnostic testing for humans, how soon is testing to be established?

- ☐ within one year
- ☐ 2 - 5 years
- ☐ 6 - 10 years
- ☐ Not sure

24) (a) Do you conduct animal rabies diagnostic testing in your country?

(b) If yes, which test(s) do you implement?

- ☐ DFA
- ☐ PCR
- ☐ Histopathology
- ☐ Mouse inoculation
- ☐ Serology
- ☐ dRIT
- ☐ Other

(c) If yes, do you receive animal samples from other countries for testing?

(d) (i) If no, do you send animal samples to laboratories in other countries for testing?

(ii) Which laboratories (including country) do you send animal samples to for testing?
(indicate type of samples)

(e) (i) If no, does your country have any immediate plans to set up rabies diagnostic testing for animals?

(ii) If your country plans to set up rabies diagnostic testing for animals, how soon is testing to be established?

- ☐ within one year
- ☐ 2 - 5 years
- ☐ 6 - 10 years
- ☐ Not sure

IMPORT HEALTH STANDARDS

25) (a) Does your country have import health restrictions related to rabies?

(b) What species do these restrictions apply?

- ☐ Dog
- ☐ Cat
- ☐ Wild Carnivores
- ☐ Other

(c) List the countries to which these restrictions currently apply:

(d) Is rabies vaccination a requirement for entry?

(e) Is rabies titer (serological) testing a requirement?

(f) (i) If rabies vaccination and titer testing are required, what is the minimum time allowed between rabies vaccination and titer testing?

- ☐ Not specified
- ☐ One month
- ☐ Other

(ii) If rabies vaccination and titer testing are required, what is the maximum time allowed between serological testing and importation?

- ☐ Not specified
- ☐ One year
- ☐ Other

(g) List the countries that are currently exempt from these restrictions:

26) (a) Do you have protocols in place to deal with animals that are imported into your country without meeting the import health requirements for rabies?

(b) If yes, what do these protocols involve?

- ☐ Re-exportation/ Refusal of entry
- ☐ Euthanasia
- ☐ Other

(c) (i) If these protocols exist do they differ between importation from OIE listed rabies endemic and rabies free countries?

(ii) If yes, what are the differences?

27) (a) Do you have a quarantine station for animals being imported into your country?

(b) If yes, what species of animals are housed?

- ☐ Dog
- ☐ Cat
- ☐ Equine
- ☐ Ruminant
- ☐ Avian
- ☐ Other

(c) (i) What is the minimum quarantine period allowed?

(ii) What animal species does this pertain to?

(iii) What are the pre-requisite conditions for the minimum period of quarantine?

(d) (i) What is the maximum quarantine period implemented?

(ii) What animal species does this pertain to?

(iii) What is the rationale behind the maximum quarantine period?

Once you have completed the questionnaire, please ensure that you are connected to the internet and click submit to send your response.

THANK YOU FOR YOUR COOPERATION

Submit
