# Supplementary material for: Rabies in the Caribbean: A Situational Analysis and Historic Review
Source: Trop Med Infect Dis. 2018 Aug 20;3(3):89. doi: 10.3390/tropicalmed3030089 (PMC6160905; doi:10.3390/tropicalmed3030089)
Supplement: Supplementary file 1 [file tropicalmed-03-00089-s001.zip › tropicalmed-343539-supplementary/tropicalmed-343539 supplementary.pdf]

Rabies in the Caribbean: a situational analysis and historic review

Janine Seetahal *, Alexandra Vokaty, Marco Vigilato, Christine Carrington, Jennifer Pradel, Bowen Louison, Astrid Van Sauers, Rohini Roopnarine, Jusayma Gonzalez Arrebato, Max Millien, Colin James, Charles Rupprecht

Table S1. Rabies disease status and national notification status of Caribbean countries and territories surveyed.

Location	Rabies status reported on survey	Notifiable disease in animals	Notifiable disease in humans
Aruba	Non-endemic	No	No
Barbados	Non-endemic	Yes	Yes
Belize	Endemic	Yes	Yes
Bonaire	Non-endemic	Yes	Yes
Bermuda	Non-endemic	Yes	Yes
British Virgin Islands	Non-endemic	No	No
Cayman islands	Non-endemic	Yes	Yes
Cuba	Endemic	Yes	Yes
Curacao	Non-endemic	Yes	Yes
Dominica	Non-endemic	Yes	No
Dominican Republic	Endemic	Yes	Yes
French Guiana	Endemic	Yes	Yes
Grenada	Endemic	Yes	Yes
Guadeloupe	Non-endemic	Yes	Yes
Guyana	Endemic	Yes	Yes
Haiti	Endemic	Yes	Yes
Jamaica	Non-endemic	Yes	No
Nevis (St. Kitts)	Non-endemic	Yes	No
Martinique	Non-endemic	Yes	Yes
Montserrat	Non-endemic	Yes	Yes
Puerto Rico	Endemic	Yes	Yes
St. Eustatius	Non-endemic	Yes	Yes
St. Kitts	Non-endemic	Yes	No
St. Lucia	Non-endemic	Yes	Yes
St. Maarten	Non-endemic	No	Yes
St. Vincent and the Grenadines	Non-endemic	Yes	Yes
Suriname	Endemic	Yes	Yes
Trinidad and Tobago	Endemic	Yes	Yes
Turks and Caicos	Non-endemic	Yes	Yes
US Virgin Islands	Non-endemic	Yes	Yes

Table S2. Mandatory reporting of rabies virus exposure incidents in humans and animals.

Location	Human incidents	Species involved	Animal incidents	Species involved
Belize	Yes	Dog, bat, fox and any wild animal	No	n.a.
Cuba	Yes	Dog, bat, mongoose, rodents, cat, pig	No	n.a.
Dominican Republic	Yes	Dog, mongoose	Yes	Dog, bat, mongoose
French Guiana	Yes	Dog, bat, mongoose, all mammals	Yes	Dog, bat, mongoose, all mammals
Grenada	Yes	Dog, mongoose, ruminants, cat	Yes	Dog, mongoose, cat
Guyana	n.r.	n.r.	Yes	Bat
Haiti	Yes	Dog	Yes	Dog
Puerto Rico	Yes	All mammals	Yes	All mammals
Suriname	No	n.a.	No	n.a.
Trinidad and Tobago	No	n.a.	Yes	Not specified
Aruba	No	n.a.	No	n.a.
Barbados	No	n.a.	No	n.a.
Bonaire	Yes	Dog	Yes	Dog
Bermuda	Yes	Dog, cat	No	n.a.
British Virgin Islands	No	n.a.	No	n.a.
Cayman Islands	No	n.a.	No	n.a.
Dominica	n.r.	n.r.	n.r.	n.r.
St. Maarten	Yes	Dog	No	n.a.
Guadeloupe	Yes	Dog, bat, cat, cattle	No	n.a.
Jamaica	No	n.a.	No	n.a.
Turks and Caicos	No	n.a.	Yes	Dog
US Virgin Islands	Yes	Dog, mongoose	Yes	Dog, mongoose
Montserrat	Yes	Dog	No	n.a.
Curacao	No	n.a.	Yes	Dog, bat
Martinique	Yes	All mammals	Yes	All mammals
Sint Eustatius	Yes	Dog	Yes	Dog
St. Kitts	No	n.a.	No	n.a.
Nevis (St. Kitts)	No	n.a.	No	n.a.
St. Lucia	No	n.a.	No	n.a.
St. Vincent and the Grenadines	No	n.a.	No	n.a.

n.r. (no response), **n.a.** (not applicable)

Table S3. Bat species richness and species identified as rabies positive in the Caribbean

Location	Bat species richness (No. of species)	Bat species positive for rabies
Belize	70 [1]	*Myotis fortidens* (Cinnamon myotis bat), *Myotis nigricans* (Black myotis bat), *Artibeus lituratus* (Great fruit-eating bat), *Artibeus jamaicensis* (Jamaican fruit-eating bat), *Phyllostomus discolour* (Pale spear-nosed bat), *Molossus molossus* (Pallas's mastiff bat), *Molossus sinaloae* (Sinaloan mastiff bat), *Desmodus rotundus* (Common vampire bat) [2]
Cuba	26 [3]	*Eptesicus fuscus* (Big brown bat), *Eumops glaucinus* (Wagner's bonneted bat), *Artibeus jamaicensis* (Great fruit-eating bat), *Molossus molossus* (Pallas's mastiff bat) [2,4,5]
French Guiana	102 [6,7]	*Desmodus rotundus* (Common vampire bat) [8]
Grenada	12 [9]	*Artibeus jamaicensis* (Great fruit-eating bat), *Molossus molossus* (Pallas's mastiff bat) [10,11]
Guyana	121 [6,7]	No surveillance
Hispaniola (Haiti and Dominican Republic)	18 [12]	*Tadarida brasiliensis* (Brazilian free-tailed bat)* [13]
Puerto Rico	13 [14]	None identified to date [14,15]
Suriname	96 [7]	No surveillance
Trinidad	69 [16,17]	*Desmodus rotundus* (Common vampire bat), *Diaemus youngi* (White winged vampire bat), *Carollia perspicillata* (Seba's short-tailed bat), *Artibeus lituratus* (Great fruit-eating bat), *Artibeus jamaicensis* (Jamaican fruit-eating bat), *Molossus molossus* (Pallas's mastiff bat), *Diclidurus albus* (Northern ghost bat), *Pteronotus davyi* (Davy's naked-backed bat), *Pteronotus parnellii* (Parnell's mustached bat) [2,18]

* isolated in the Dominican Republic

References

1. Fenton, M.B.; Bernard, E.; Hollis, L.; Johnston, D.S.; Lausen, C.L.; Ratcliffe, J.M.; Riskin, D.K.; Taylor, J.R.; Zigouris, J. The bat fauna of lamanai, belize: Roosts and tropical roles *Journal of Tropical Ecology* **2001**, *17*, 511-524.
2. Constantine, D.G. *Bat rabies and other lyssavirus infections*. United States Geological Survey National Wildlife Health Center: Reston, Virginia, USA, 2009; Vol. 1329.
3. Willig, M.R.; Presley, S.J.; Bloch, C.P.; Genoways, H.H. Macroecology of caribbean bats: Effects of area, elevation, latitude and hurricane-induced disturbance. In *Island bats: Evolution, ecology and conservation*, Fleming, T.H.; Racey, P.A., Eds. The University of Chicago Press Ltd: Chicago, USA, 2009; pp 216-264.

4. Nadin Davis, S.A.; Torres, G.; Ribas, M., .; Guzman, M., . ; Cruz De La Paz, R.; Morales, M.; Wandeler, A.I. A molecular epidemiological study of rabies in cuba. *Epidemiol Infect* **2006**, *134*, 1313-1324.
5. Cordero, Y.; de los Angeles, R.M.; Beatriz, C.H.; Cintra, Y.; Tejero, Y.; Gonzalez, J.; Daileny, P.; Castilho, J. In *Epidemiology and molecular characterization of rabies in cuba from 2003 to 2016*, XXVIII Rabies in the Americas, Calgary, Alberta, Canada, 2017; Calgary, Alberta, Canada, p 17.
6. Lim, B.K.; Emngstrom, M.D. Species diversity of bats (mammalia: Chiropter) in iwokrama forest, guyana, and the guianan subregion: Implications for conservation *Biodiversity & Conservation* **2001**, *10*, 613-657.
7. Shapley, R.L.; Wilson, D.E.; Warren, A.N.; Barnett, A.A. Bats of the potaro plateau region, western guyana. *Mammalia* **2005**, *69*, 375-394.
8. Berger, F.; Desplanches, N.; Baillargeaux, S.; Joubert, M.; Miller, M.; Ribadeau-Dumas, F.; Spiegel, A.; Bourhy, H. Rabies risk: Difficulties encountered during management of grouped cases of bat bites in 2 isolated villages in french guiana. *PLoS Negl Trop Dis* **2013**, *7*.
9. Genoways, H.H.; Phillips, C.J.; Baker, R.J. Bats of the antillean island of grenada: A new zoogeographic perspective. *Mammalogy Papers: University of Nebraska State Museum* **1998**, *98*.
10. Price, J.L.; Everard, C.O.R. Rabies virus and antibody in bats in grenada and trinidad. *Journal of Wildlife Diseases* **1977**, 131-134.
11. Everard, C.O.R.; Murray, D.; Gilbert, P.K. Rabies in grenada. *Transactions of the Royal Society of Tropical Medicine and Hygiene* **1972**, *66*, 878-888.
12. Flanders, J.; Yohe, L.; Rossiter, S.; Davalos, L.M. Field key to the bats of hispanola. **2014**.
13. Organizacion Panamericana de la Salud (OPS). *Republica dominicana: Elimination of dog-transmitted rabies in latin america: Situation analysis*; Washington, 2004; pp 58-59.
14. Gannon, M.R.; Kurta, A.; Rodriguez-Duran, A.; Willig, M.R. *Bats of puerto rico: An island focus and an caribbean perspecitive*. Texas Tech University Press: Lubbock, Texas, USA, 2005.
15. Everard, C.O.R.; Everard, J.D. Mongoose rabies in the caribbean. *Annals of NY Acad Sci* **1992**, *653*, 356-366.
16. Gomes, G.A.; Reid, F.A. *Bats of trinidad and tobago: A field guide and natural history*. Trinibats: Trinidad, 2015.
17. Moratelli, R.; Wilson, D.E.; Novaes, R.L.M.; Helgen, K.M.; Gutierrez, E.E. Caribbean myotis (chiroptera, vespertilionidae), with description of a new species from trinidad and tobago. *Journal of Mammology* **2017**, 1-15.
18. Goodwin, G.G.; Greenhall, A. *A review of the bats of trinidad and tobago: Descriptions, rabies infection and ecology*. 1961; Vol. 122.
